# Host immunity in the protective response to vaccination with heat-killed *Burkholderia mallei*

**DOI:** 10.1186/1471-2172-9-55

**Published:** 2008-09-29

**Authors:** Gregory C Whitlock, Roman A Lukaszewski, Barbara M Judy, Slobodan Paessler, Alfredo G Torres, D Mark Estes

**Affiliations:** 1 Department of Microbiology and Immunology, University of Texas Medical Branch, Galveston, Texas, USA; 2 Department of Clinical Laboratory Sciences, University of Texas Medical Branch, Galveston, Texas, USA; 3 Department of Pediatrics, University of Texas Medical Branch, Galveston, Texas, USA; 4 Department of Pathology, University of Texas Medical Branch, Galveston, Texas, USA; 5 Sealy Center for Vaccine Development, University of Texas Medical Branch, Galveston, Texas, USA; 6 DSTL Biomedical Sciences, Porton Down, Salisbury, Wiltshire, SP4 0JQ, UK

## Abstract

**Background:**

We performed initial cell, cytokine and complement depletion studies to investigate the possible role of these effectors in response to vaccination with heat-killed *Burkholderia mallei *in a susceptible BALB/c mouse model of infection.

**Results:**

While protection with heat-killed bacilli did not result in sterilizing immunity, limited protection was afforded against an otherwise lethal infection and provided insight into potential host protective mechanisms. Our results demonstrated that mice depleted of either B cells, TNF-α or IFN-γ exhibited decreased survival rates, indicating a role for these effectors in obtaining partial protection from a lethal challenge by the intraperitoneal route. Additionally, complement depletion had no effect on immunoglobulin production when compared to non-complement depleted controls infected intranasally.

**Conclusion:**

The data provide a basis for future studies of protection via vaccination using either subunit or whole-organism vaccine preparations from lethal infection in the experimental BALB/c mouse model. The results of this study demonstrate participation of B220^+ ^cells and pro-inflammatory cytokines IFN-γ and TNF-α in protection following HK vaccination.

## Background

*Burkholderia mallei*, the etiologic agent of glanders, is a gram-negative, capsulated, non-motile, facultative intracelluar bacterium. Most known members of the *Burkholderiaceae *are resident in the soil; however, *B. mallei *is thought to be an obligate mammalian pathogen. Horses are highly susceptible to infection and considered the natural reservoir for infection, although mules and donkeys are also susceptible [1]. Clinically, glanders in solipeds can present as either a chronic (horses) or acute (mules and donkeys) form. Naturally acquired human infection with *B. mallei*, although not seen in the United States since 1945, has occurred rarely and sporadically among laboratory workers and those in direct contact with infected animals [2]. However, glanders is endemic among domestic animals in Africa, Asia, the Middle East, and Central and South America. The course of infection is dependent on the route of exposure. Direct contact with the skin can lead to a systemic infection. Inhalation of aerosol or dust containing *B. mallei *can lead to septicemic, pulmonary, or chronic infections of the muscle, liver and spleen. The disease has a 95% case fatality rate for untreated septicemic infections and a 50% case fatality rate in antibiotic-treated individuals [3].

There is no human or animal vaccine available for glanders, and development of a partial or fully protective adaptive host response to the organism has not been well-defined. Previous studies with *B. mallei *and the host response have shown that a mixed immune response consisting of both Th_1 _and Th_2_-associated cytokines with a predominant IgG1 subclass does not correlate with protection [4]. Additional studies with passive transfer of monoclonal antibodies specific for *B. mallei *have correlated with early protection from infection [5]. Recent studies have also shown the Th_1 _cytokine IL-12 to mediate partial protection to non-viable *B. mallei*-vaccinated mice [6]. Thus, full correlates of protection mediated by the adaptive immune system against *B. mallei *remain to be fully elucidated.

In this series of studies, we sought to address the impact of depletion of the major effector lymphoid cell populations (B220^+ ^B cells, CD4^+ ^or CD8^+ ^T cells) and key pro-inflammatory/Type 1 cytokines (IFN-γ or TNF-α) on survival in BALB/c mice vaccinated with heat killed (HK) bacilli followed by an intraperitoneal (i.p.) challenge with live organism. In addition, studies investigating the effect of complement on opsonization of organism and antibody production were assessed. Heat killed bacteria were used as a model of vaccination to allow evaluation of *B. mallei *specific immune responses. The results of this study demonstrate participation of B220^+ ^cells and pro-inflammatory cytokines IFN-γ and TNF-α in protection following HK vaccination.

## Results

### Heat-killed *B. mallei *vaccination mediates partial protection from lethal challenge

**Figure 1:**
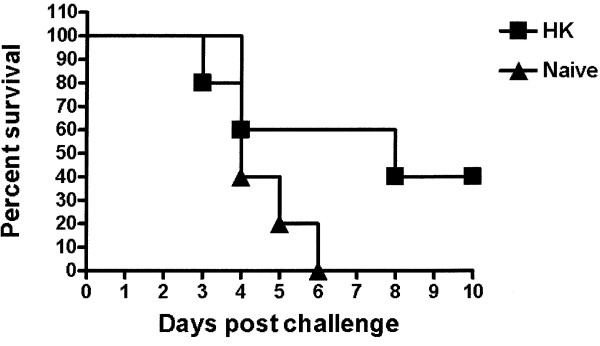
**Percentage of survival in heat-killed vaccinated BALB/c mice**. BALB/c mice were vaccinated with 1 × 10^5 ^CFU/100 μl of HK *B. mallei *by intraperitoneal injection. Two weeks post HK vaccination, mice were injected i.p. with 2 × 10^7 ^CFU/100 μl of live *B. mallei *(~20 LD_50_). HK vaccination resulted in a 40% survival rate for HK-vaccinated mice with a mean survival time (MST) of 8 days (p = 0.1526). Immunologically naïve mice demonstrated 100% mortality by day 6. Data are representative of 2 independent experiments.

To begin to address this issue in an animal model of acute infection, we established that immunologically naive BALB/c mice challenged i.p. with 2 × 10^7 ^CFU resulted in death by day 4–6, while i.p. immunization with 1 × 10^5 ^heat killed (HK) bacteria provided partial protection against a subsequent challenge. Two independent experiments resulted in similar findings of 40% survival for HK-vaccinated mice with a mean survival time (MST) of 8 days versus 4 days in naïve mice (Fig. 1). The administration of vaccines for *B. mallei *during an outbreak would mandate relatively rapid onset of protection for human or veterinary use. Based on non-routine use and vaccine implementation in the course of an outbreak, a 14 day window was chosen for assessment of protection. Our results indicate that HK vaccination can afford partial protection to an otherwise lethal challenge of *B. mallei *by the i.p. route.

### Effects of cell depletion on HK-vaccinated survival

**Figure 2:**
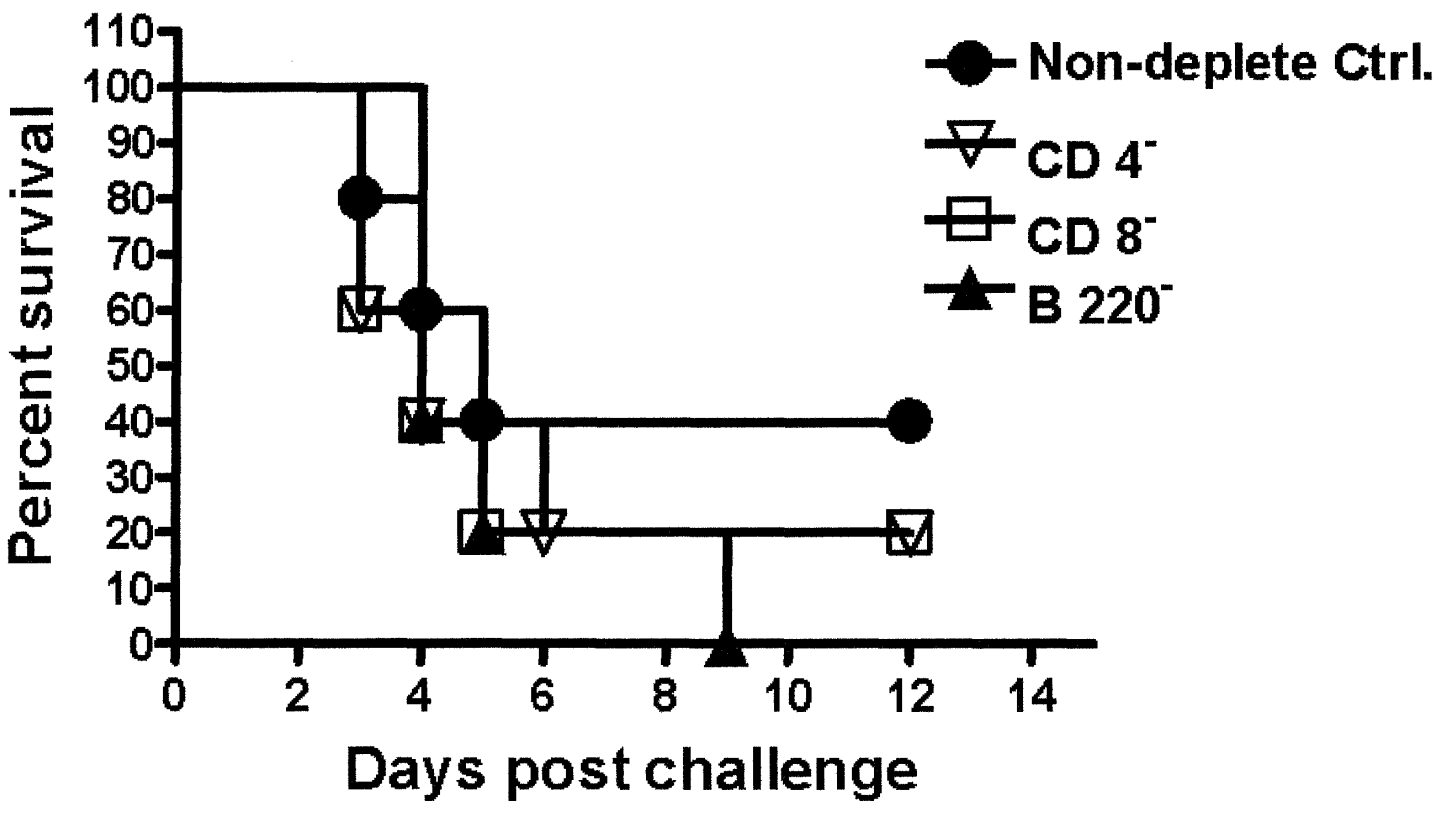
**Percentage of survival among CD8, CD4, or B220 cell-depleted, HK-vaccinated BALB/c mice**. Following cell depletion, mice were challenged with 2 × 10^7 ^CFU *B. mallei *(n = 5 per group). CD4 (p = 0.5417) and CD8 (p = 0.4684)-depleted mice demonstrated a 50% decreased survival rate compared to that of the isotype control. B220-depleted mice resulted in 100% decreased survival (p = 0.3418) compared to that in non-depleted isotype control mice.

**Figure 3:**
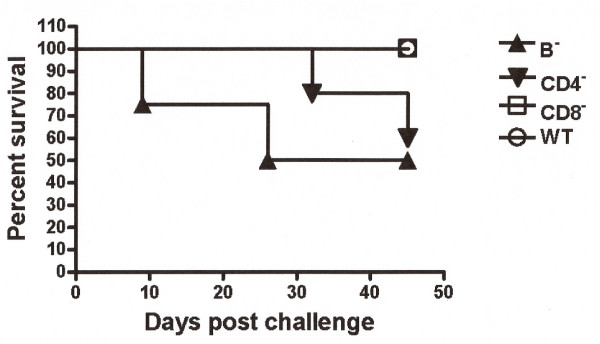
**Percentage of survival among C57BL/6 B-cell (μMT), CD4 T-cell (CD4^-/-^) and CD8 T-cell (CD8^-/-^)-deficient, HK-vaccinated mice**. Two weeks post vaccination, mice were challenged with 2 × 10^7 ^CFU/100 μl of live *B. mallei *by intraperitoneal injection. B-cell-deficient mice demonstrated a 50% decreased survival (p = 0.0888) compared to that of the wild-type mice with a MST of 35.5 days (n = 6). CD4^-/- ^and CD8^-/- ^mice resulted in 60% (p = 0.1343) and 0% reduced survival, respectively (n = 5).

To dissect the cellular basis for protection mediated by HK vaccination, 13 days after immunization with HK bacteria (day -1), and at day of challenge, mice were dosed with antibodies to deplete CD4^+^, CD8^+ ^or B220^+ ^cells. Antibody depletion of CD4^+^, CD8^+^, or B220^+ ^cells in these mice was confirmed by flow cytometric analysis with depletion efficiencies for CD4, CD8, and B220 populations at 99.7%, 96%, and 95%, respectively, relative to mice treated with isotype control monoclonal antibodies (data not shown). Our results demonstrated decreased survival rates in B220 (p = 0.3418), CD4^+ ^(p = 0.5417) and CD8^+ ^(p = 0.4684) antibody depleted mice, compared to isotype control antibody, a finding that indicated a possible role for vaccine induced antibody production. When challenged with 2 × 10^7 ^CFU/mouse by the i.p. route, loss of T cells resulted in reduced survival (50%) relative to the non-specific isotype control (Fig. 2). In contrast to the loss of T cells, depletion of B220^+ ^cells resulted in 100% mortality relative to the non-specific isotype control (Fig. 2). To further evaluate the necessity of these effector cells in providing protection following HK vaccination, relatively resistant C57BL/6 mice, deficient in mature B-cells (μMT), CD4 T-cells (CD4^-/-^) or CD8 T-cells (CD8^-/-^) were subjected to an identical HK vaccination and challenge regimen. Mature, B-cell-deficient mice demonstrated a 50% decreased survival (p = 0.0888) compared to the wild-type mice with an MST of 35.5 days (Fig. 3). CD4^-/- ^and CD8^-/- ^mice exhibited a 60% (p = 0.1343) and 0% reduced survival, respectively (Fig. 3).

### Effects of cytokine depletion on HK vaccination

**Figure 4:**
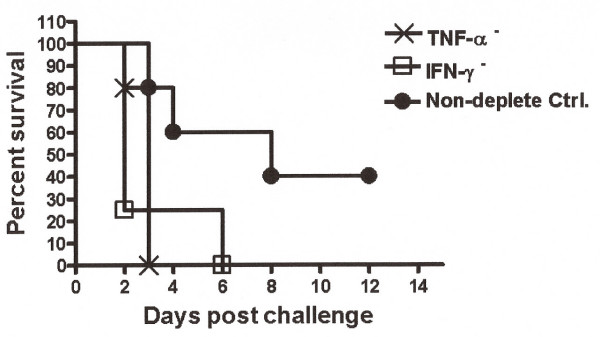
**Percentage of survival in IFN-γ or TNF-α depleted, HK-vaccinated mice**. Following individual cytokine depletions, mice were challenged with 2 × 10^7 ^CFU *B. mallei *by intraperitoneal injection (n = 5 per group). At day 6 post infection, IFN-γ depleted mice demonstrated a 100% mortality (p = 0.0446) compared to that of the isotype control. At day 3 post infection, TNF-α-depleted mice demonstrated 100% mortality (p = 0.0145) compared to that of the isotype control. Results are representative of two experiments with the same group sizes.

**Figure 5:**
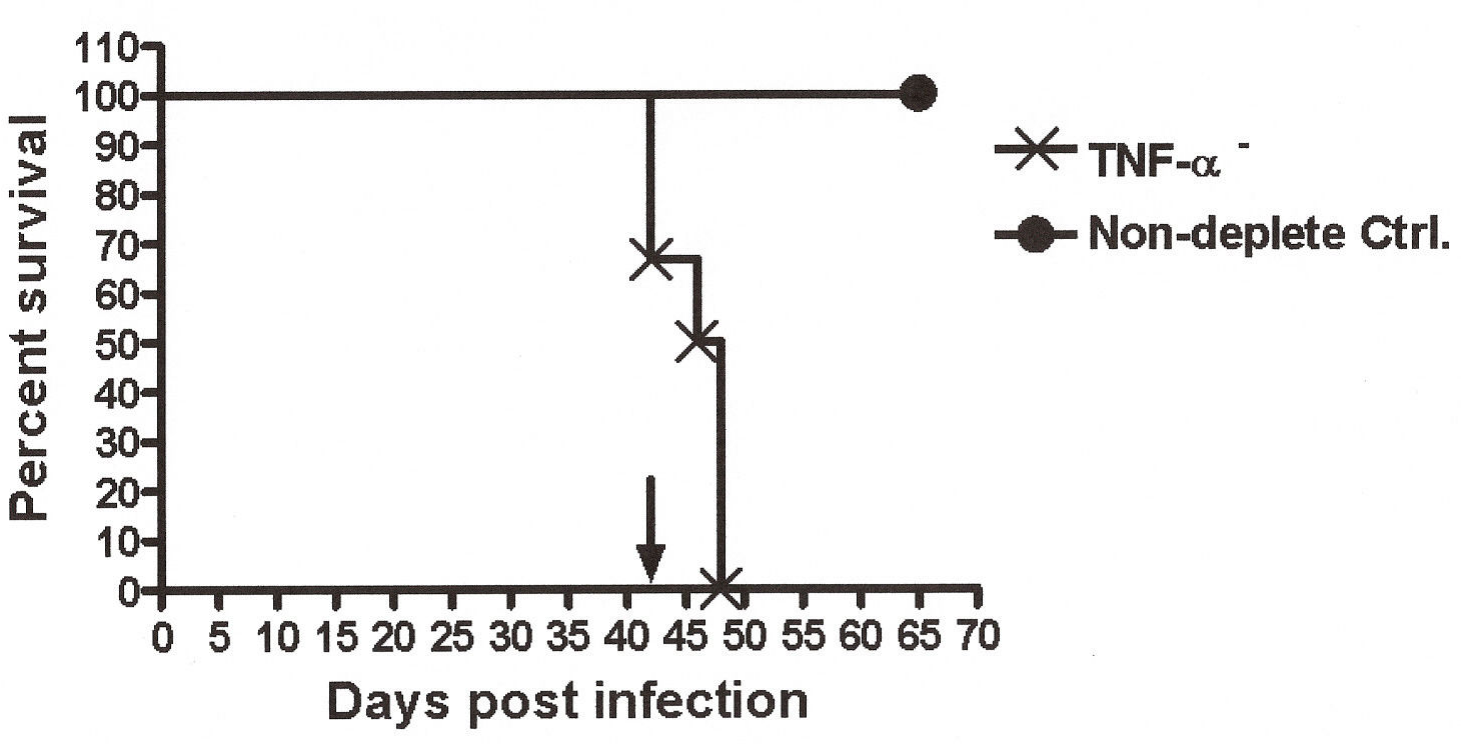
**TNF-α impact on chronic *B. mallei *infection**. BALB/c mice challenged i.p. with 1 × 10^6 ^CFU *B. mallei *were depleted of TNF-α (n = 6) or antibody control (n = 5) at day 42 post infection. Rapid mortality was observed at 7 days post TNF-α depletion (p = 0.0023).

Similar studies were performed to determine the role of IFN-γ or TNF-α in acute infection in BALB/c mice immunized with HK bacteria. Six hours before challenge, mice were dosed with antibodies that neutralize IFN-γ or TNF-α. Individual depletion of either TNF-α (p = 0.0145) or IFN-γ (p = 0.0446) resulted in 100% mortality with an MST of 3 and 2 days, respectively, compared to the HK-vaccinated isotype control mice (Fig. 4). In contrast, 40% of HK-vaccinated, isotype control mice survived to at least 12 days post-challenge (Fig 4). To further evaluate the host TNF-α response during an established *B. mallei *chronic infection, we infected 12 BALB/c mice by the i.p. route with 1 × 10^6 ^CFU *B. mallei*. One animal was terminally ill on day 37 post-infection. On day 42 post-infection, the remaining 11 mice were dosed with either anti-TNF-α (n = 6), or control mAb (AFRC Mac 49) (n = 5). No further deaths were observed in the control mAb-treated mice. Rapid mortality was observed in the anti-TNF-α-treated group, with all mice dying within 7 days of treatment (p = 0.0023) relative to the isotype-treated controls (Fig. 5).

### J774A.1 uptake of serum treated *B. mallei*

**Figure 6:**
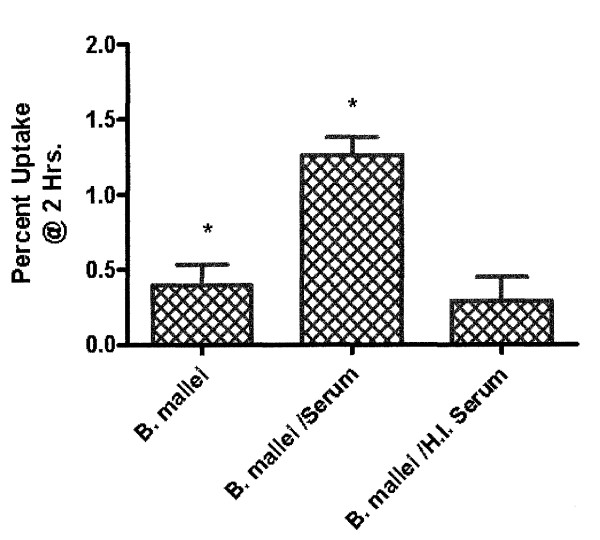
**In vitro uptake of *B. mallei***. J774A.1 cells were incubated with *B. mallei *(MOI 10:1) alone, supplemented with either 2% mouse serum or heat inactivated (H.I.) (56°C 30 min.) mouse serum. Experiment performed in triplicate with data expressed as mean ± S.D. *p = 0.0082.

Complement mediated uptake assays were performed to evaluate opsonization. Results indicated enhanced bacterial uptake in J774A.1 phagocytes inoculated with serum treated *B. mallei *(p = .0082), compared to *B. mallei *alone, while heat-inactivated serum produced uptake percentages similar to those prior to serum addition (Fig. 6). Taken together, these results imply an active role for complement components in the uptake of organism by macrophages.

### Immunoglobulin production in HK vaccinated BALB/c mice

We further characterized the ability of HK vaccination to induce a predominant IgG isotype by determining IgG2a/IgG1 ratios in i.p. and i.n. vaccinated BALB/c mice. Pre (day 14 post vaccination) and post (day 2 post infection) exposure serum samples were obtained and evaluated for IgG isotype concentrations (Table 1). No appreciable differences in IgG pre-exposure levels were seen when comparing i.n. to i.p. vaccination. In addition, cobra venom factor-treated animals showed no significant differences to non-cobra venom factor-treated animals in IgG pre-exposure (challenge) levels. Conversely, isotype switching in the cobra venom factor treated animals was enhanced in post-exposure serum IgG2a (Table 1).

**Table 1:** Murine immune responses to HK vaccination

**Vaccine**	**Pre-exposure**			**Post-exposure**		
	IgG2a	IgG1	Ratio	IgG2a	IgG1	Ratio

HK i.n.	0.00	0.07 + .005	0.00	0.88 + .031	1.46 + .027	0.60
HK i.p.	0.01 + .012	0.23 + .015	0.04	1.04 + .037	0.92 + .0005	1.13
HK i.p. CVF	0.01 + .002	0.13 + .004	0.07	1.42 + .020	1.02 + .002	1.39
None	0.00	0.00	0.00	0.86 + .025	1.01 + .002	0.85

Samples were tested in duplicate and optical densities (OD) read at 450 nm. Values are reported as mean (± S.E.M.). n = 5 for pre-exposure and n = 2 for post exposure. CVF; cobra venom factor.

## Discussion

Recent studies have shown a key role in protection from lethal challenge for IFN-γ in non-vaccinated mice from either NK and/or NKT cells following experimental exposure to *B. mallei *and *B. pseudomallei *[7,8]. A similar protective role in the innate response to infection has been demonstrated for TNF-α in *B. pseudomallei *infection [8]. The studies presented here are consistent with the essential role of these factors in the relative levels of protection conferred by vaccination with heat-killed *B. pseudomallei *and would appear to be viable early markers for protection from lethal acute infection [9]. Currently, there are no fully protective vaccines against *B. mallei *or *B. pseudomallei *in a murine model, particularly for the sensitive BALB/c versus C57BL6 models. Previous studies have also demonstrated that both the humoral and cell-mediated arms are essential for protection from *B. pseudomallei *infection [10]. Thus, loss or reduction of TNF-α and IFN-γ levels result in significantly reduced survival rates, substantiating previous reports of the role of these factors in protection against *B. mallei *[7]. Moreover, we demonstrate a role for sustained TNF-α production in the maintenance of host survival throughout the course of *B. mallei *infection. Mice with an established *B. mallei *chronic infection rapidly lost the ability to control the growth of the bacillus upon neutralization of TNF-α. This would suggest a potential role for TNF-α in the maintenance of productive granulomas which may limit the spread of bacteria in chronically infected hosts, or, alternatively, in direct or indirect microbicidal or bacteriostatic activities at the sites of infection. Additional studies are underway to determine more precisely the role of TNF-α in host protection to *B. mallei*.

Multiple innate and adaptive cell types may contribute to the production of IFN-γ in response to infection with *B. mallei *following vaccination. Our results with individual depletion of CD4^+ ^and CD8^+ ^T cells suggests that both cell types may compensate for the functional loss of the other effector cell type in the production of this key cytokine. The effector role for IFN-γ in mediating protection against *B. mallei *may include both immunoregulatory and non-regulatory functions. Regardless, the requirement of IFN-γ, as demonstrated by administration of neutralizing antibody prior to infection, indicates that stimulation of IFN-γ response is a desirable goal for a *B. mallei *vaccine.

Similarly, B220-positive cells appear to play a role in protection following vaccination with heat-killed *B. mallei*. Interestingly, this protective immunity, occurring in other intracellular pathogens, is not exclusively dependent on B cells [11]. Passive protection has been demonstrated against acute *Burkholderia *infection by monoclonal antibodies [5,12]. Protection against *B. pseudomallei *infection by anti-LPS, capsular polysaccharide and proteins has been short-lived, suggesting that antibody production offers limited protection in the initial stages of infection by an as-yet-undefined mechanism [12]. We have shown that following depletion of B220^+ ^cells, survival rates decreased as much as 100% relative to non-depleted controls and individual CD4/CD8-depleted mice via the intraperitoneal route. Results from C57BL/6 mice deficient in mature B-cells (μMT), CD4 T-cells (CD4^-/-^) or CD8 T-cells (CD8^-/-^) substantiate the requirement for B-cell involvement by evidence of μMT and CD4^-/- ^decreased survival. The lack of an effective CTL response to vaccination did not appear to alter survival in what would appear to be a CD4/B-cell (humoral)-driven response. In CD4-deficient mice, we have the additional potential variable that a CD4-dependent antibody response might also be inhibited during the vaccination phase relative to mice treated with antibody immediately prior to and during the early phases of infection. Although not statistically significant, we did observe a decrease in survival in μMT (mature B cell) deficient mice as early as day 9 post challenge, whereas CD4-deficient mice produced similar results at day 32 post challenge, indicating a role for B cells independent of CD4 T cell help, perhaps through a T-independent mechanism of antibody production. Although CD8^-/- ^C57BL/6 demonstrated no decreased survival in our HK-vaccinated model, a lack of potential endogenous protein production by HK *B. mallei *may have contributed to limited MHC-I presentation.

Complement associated studies revealed increased J774A.1 uptake of serum-treated *B. mallei*. Complement-mediated uptake studies of *B. pseudomallei *by polymorphonuclear leukocytes (PMNs) suggest that capsule production contributes to resistance of phagocytosis by reducing C3b bacterial deposition [13]. Previous studies have demonstrated that a polysaccharide capsule is present in *B. mallei*, [14,15] although in the present study enhanced uptake with serum-treated *B. mallei *was observed. Intracellular survival assays of complement mediated uptake of organisms were not performed in the present study, thus, the role of complement opsonization on intracellular survival is not fully resolved. Previous reports have demonstrated the ability of *B. mallei *to survive within macrophage without the aid of serum coating organisms [16]. Conversely, the idea of antibody mediated opsonization to facilitate macrophage activation and clearance of intracellular organisms may offer support to the role of B cells in an effective immune response. A possible protective mechanism may include HK vaccination induced production of opsonizing antibodies which may aid in complement mediated uptake, thereby limiting the initial bacterial threshold below a lethal level.

Immunoglobulin responses to HK vaccination resulted in modest levels of IgG1 following 2 weeks post vaccination, while post-exposure levels were indicative of efficient class switching to a favorable IgG2a isotype. Importantly, cobra venom factor treatment of animals at time of vaccination did not alter their ability to produce immunoglobulin. In fact, cobra venom factor treated animals resulted in higher IgG2a levels when compared to non-treated. Complement activation can modulate both the primary and secondary immune responses and has been shown to enhance secondary immune responses to vaccination [17]. The current results suggest that cobra venom factor treatment may affect the modulation of the immune response to *B. mallei *infection through B cell activation and/or memory B cell generation.

## Conclusion

In summary, our results provide a basis for future studies of protection via vaccination using either subunit or whole-organism vaccine preparations from lethal infection in the experimental BALB/c mouse model. Understanding and defining the role of B cells in adaptive *B. mallei *immunity will likely be fundamental to the design of an efficacious vaccine and important goals of future research.

## Methods

### Bacterial strain and mice

*B. mallei *strain ATCC 23344 (China 7) was cultured on Luria-Bertani agar supplemented with 4% glycerol (LB+4%G) agar plates for 48 h at 37°C. Isolated colonies were sub-cultured to LB+4%G broth, and cultures were incubated at 37°C until optical density readings at 600 nm (OD_600_) reached an exponential phase of growth. Bacteria were pelleted by centrifugation, washed and re-suspended in sterile 1× phosphate-buffered saline (PBS, pH 7.4) to obtain the desired CFU/ml. To obtain HK inoculums, bacterial suspensions were incubated at 85°C for 3 h and stored at 4°C until use. The absence of live *B. mallei *organisms in the HK preparations was confirmed after plating 10% of the total inoculums (v/v) and incubating these at 37°C for 48 h. All procedures were performed under a class II biosafety cabinet in a biosafety level 3 laboratory. Female, 6- to 8-week-old, BALB/c mice (n = 5–7) were obtained from Harlan Sprague Dawley, Inc. (Indianapolis, Indiana). Female, 6- to 8-week-old, C57BL/6 mice deficient in mature B-cells (μMT), CD4 T-cells (CD4-/-) and CD8 T-cells (CD8-/-) and wild-type mice were obtained from The Jackson Laboratory (Bar Harbor, Maine).

### Vaccination and challenge

BALB/c and C57BL/6 mice were grouped and vaccinated with 0.5 μg of HK *B. mallei *(without adjuvant) by i.p. injection using a 25-gauge syringe. Two weeks post HK vaccination mice were injected i.p. with 2 × 10^7 ^CFU/100 μl of live *B. mallei *(~20 LD_50_) [18]. Complement depleted animals were challenged with 2.5 × 10^4 ^CFU/50 μl (~0.25 LD_50_) by intranasal (i.n.) route. Aliquots from the inoculums were plated to confirm the infecting dose. All procedures and animal protocols used in this study were approved by the Biosafety and IACUC committees at UTMB and conducted in either BSL-3 or ABSL-3 laboratories.

### Cell and cytokine depletions

Acute *in vivo *cell/cytokine depletion was performed with monoclonal rat anti-mouse CD4 (GK1.5), CD8α (53-6.7) or B220 (RA3-6B2) obtained from R&D Systems, Inc. (Minneapolis, MN) by methods similar to those we have previously described [19]. Functional grade purified rat anti-mouse interferon-gamma (IFN-γ, AN-18) was obtained from eBioscience (San Diego, CA) and purified anti-mouse tumor necrosis factor (TNF-α, MP6-XT3) from BD Pharmingen (San Diego, CA). IFN-γ and TNF-α antibodies were injected i.p. 6 h prior to challenge, 200 μg per mouse in 200 μl PBS or at later time points as indicated. Rat IgG isotype control was obtained from Southern Biotech (Birmingham, AL) and administered i.p. on day of challenge, 200 μg/mouse. Rat anti-mouse CD4, CD8α and B220 were injected i.p. twice, 1 day prior to challenge and on day of challenge, with an equivalent dosage sufficient to deplete T or B cells from 6 × 10^8 ^bone marrow cells per injection. The efficiency of depletion at time of infection for CD4^+^, CD8^+^, and B220^+ ^cells was confirmed by flow cytometry analysis immediately prior to infection.

### Complement depletion with cobra venom factor

Mice, six to seven per group, were vaccinated i.p. with 1 × 10^5 ^CFU of nonviable *B. mallei *cell preparation in a total volume of 0.1 ml. Two weeks later, 24 h and 1 h before challenge, complement depleted mice were treated i.p. with 12.5 units total cobra venom factor (Quidel Corporation Speciality Products, San Diego, CA) in 0.1 ml of PBS. Complement depletion was confirmed prior to challenge by micro-titer hemolytic complement activity (CH_50_) assay as previously described [20].

### *B. mallei *J774A.1 uptake assays

J774A.1 cells were seeded (5 × 10^5^) onto Corning costar 24 well plates (Corning, NY) with DMEM and incubated overnight at 37°C with 5% CO_2_. Bacterial suspensions were incubated at 37°C for 45 minutes supplemented with 2% mouse serum from Sigma-Aldrich (St. Louis, MO.), heat inactivated mouse serum (56°C 30 minutes), or bacteria alone and then added at an MOI of 10:1 to J774A.1 cells in triplicate. Inoculated wells were centrifuged at 800 g for 2 minutes and incubated for 2 hours at 37°C with 5% CO_2 _followed by a PBS wash (×2) and 2 hour incubation with 250 μg/ml kanamycin. Wells were washed twice with PBS and lysed with 0.1% Triton X-100, followed by serial 10-fold dilutions plated on LBG plates and incubated at 37°C for 2 days. Colony forming units were enumerated and uptake expressed as a percentage of initial inoculating dose ± SEM.

### Antibodies and flow cytometry

Flow cytometric analysis was performed on 0.1-ml blood samples transferred to micro centrifuge tubes containing 90 μl of acid citrate dextrose (ACD) solution. Red blood cells were lysed using ACK-lysing buffer (Biosource International, Inc., Camarillo, CA) according to the manufacturer's instruction. Antibodies used for analysis of surface markers included: FITC-conjugated rat anti-mouse CD45R/B220 (RA3-6B2, BD Pharmingen San Diego, CA) for B cells; FITC-conjugated rat anti-mouse CD8α (53-6.7) and CD4 (GK1.5, BD Pharmingen, San Diego, CA) for CD8^+ ^or CD4^+ ^cells, respectively. Samples evaluated for CD4^+ ^and CD8α^+ ^cells were also incubated with biotin-conjugated hamster anti-mouse CD3e (145-2C11) monoclonal antibody (BD Pharmingen, San Diego, CA) and subsequently with streptavidin APC Cy7. Isotype-matched, non-specific controls were assayed in parallel (BD Pharmingen, San Diego, CA). Surface staining was performed according to previously published protocols [21]. Following cell staining, the samples were fixed with 2% buffered paraformaldehyde overnight prior to analysis by flow cytometry. Samples were analyzed using a FACSCalibur flow cytometer with BD CellQuest Pro software.

### Antibody assays

Immunoglobulin subclass IgG1 and IgG2a titers in mice were determined by a whole bacterial cell ELISA performed in 96-well, Immulon 2 HB, round-bottom plates (Dynex Technologies). *B. mallei *antigen was diluted in 0.1 M carbonate buffer (pH 9.5) and 50 μl of diluted cells placed into wells. Plates were stored overnight at 4°C. The plates were washed with washing solution (1× PBS, 0.05% Tween 20), and incubated with 100 μl of blocking solution (1× PBS, 1% bovine serum albumin, 0.05% Tween 20) for 1 h at 37°C. Dilutions of mouse sera were made with blocking solution in duplicate and plates were incubated for 1 h at 37°C. Following incubation, plates were washed and 50 μl of anti-Ig-horseradish peroxidase subclass conjugate, diluted accordingly to manufacturer's instructions (Southern Biotechnology Associates, Inc. Birmingham, Ala.), was added to each well and incubated for 1 h at 37°C. After washing, 50 μl of 2,2'-azino-di-(3-ethylbenzthizoline)-6-sulfonate (ABTS) peroxidase substrate (KPL, Inc., Gaithersburg, Maryland) was added to each well and plates incubated for 25 min at room temperature. The amount of bound antibody was determined colorimetrically by absorbance at 405 nm.

### Statistical analysis

Survival curves were calculated by Kaplan Meier survival analysis with log-rank tests between groups using GraphPad Prism (V.4.03 for windows). Statistical analysis was generally performed with the paired Student's t-test. P value ≤ 0.05 was considered significant.

## Abbreviations

HK: Heat-killed; i.p.: intraperitoneal; i.n.: intranasal.

## Authors' contributions

GCW designed and conducted experiments and drafted the manuscript. BMJ carried out the immunoassays and animal work. SP provided analysis of data and contributed to design and animal work. RAL participated in the generation and analysis of chronic TNF-α data. DME conceived the study, and participated in its design and coordination and helped to draft the manuscript. AGT participated in the bacterial work and drafting of the manuscript. All authors read and approved the final manuscript.
